# Amputation rates of the lower limb by amputation level – observational study using German national hospital discharge data from 2005 to 2015

**DOI:** 10.1186/s12913-018-3759-5

**Published:** 2019-01-06

**Authors:** Melissa Spoden, Ulrike Nimptsch, Thomas Mansky

**Affiliations:** 1 Department of Structural Advancement and Quality Management in Health Care, Technische Universität Berlin, Berlin, Germany; 2 Department of Health Care Management, Technische Universität Berlin, H80, Strasse des 17. Juni 135, 10623 Berlin, Germany

**Keywords:** Lower limb amputation, Diabetes mellitus, Peripheral arterial disease, In-hospital mortality, Hospital discharge data, Reamputation

## Abstract

**Background:**

In international comparisons, rates of amputations of the lower limb are relatively high in Germany. This study aims to analyze trends in lower limb amputations over time, as well as outcomes of care concerning in-hospital mortality and reamputation rates during the same hospital stay which might indicate the quality of surgical and perioperative health care processes.

**Methods:**

This work is an observational population-based study using complete national hospital discharge data (Diagnosis-Related Group Statistics (DRG Statistics)) from 2005 to 2015. All inpatient cases with lower limb amputation were identified and stratified by eight amputation levels. Time trends of case numbers and in-hospital mortality were studied age-sex standardized. For inpatient cases with reamputation during the same hospital stay, first and last amputation levels were cross tabulated.

**Results:**

A total of 55,595 amputations of the lower limb in 2015 (52,096 in 2005) were identified. After age-sex standardization to the demographic structure of 2005, a relative decrease of − 11.1% was revealed (men − 2.6%, women − 25.0%). The stratified analysis by amputation levels showed that the decreases were induced by higher amputation levels, whereas the amputation levels of toe/foot ray after standardization still showed a relative increase of + 12.8%. In-hospital mortality of all cases with lower limb amputation fell from 19.8% in 2005 to 17.4% in 2015 (SMR 0.89 [95% CI 0.86; 0.92]). The percentage of reamputations during the same hospital stay declined from 13.2 to 10.2%.

**Conclusions:**

The number of lower limb amputations declined in Germany, however distinctly stronger in women than in men. The observed decreases of in-hospital mortality as well as of reamputation rates point to improvements in perioperative health care. Despite these indications of improvements, the distinct increase in case numbers at the level of toe/foot ray calls for additional targeted prevention efforts, especially for patients with diabetes.

**Electronic supplementary material:**

The online version of this article (10.1186/s12913-018-3759-5) contains supplementary material, which is available to authorized users.

## Background

The amputation of the lower limb is the last treatment option for critical limb ischemia after unsuccessful vascular or endovascular treatment of the affected section [1]. It is accompanied by a high risk of death and reamputation [2, 3]. Diabetes mellitus (DM) or peripheral arterial disease (PAD) are the two major diseases leading to amputations in industrial nations. Approximately 75 to 78% of all amputations of the lower limb in Germany are attributed to DM or PAD [4–6]. Both diseases are marked by a constantly increasing prevalence. DM and PAD facilitate the development of each other and by that the prevalence of both diseases comprise overlaps [1, 7–10]. From 2009 to 2015, the prevalence of DM in Germany increased from 8.9 to 9.8% [7]. In the preceding decade the number of individuals with PAD increased around 13% in high-income countries [8].

International studies have reported declining rates of major amputations and increasing rates of minor amputations [2, 6, 11–17]. The suspected drivers behind this shift from major to minor amputations are improvements in preventive health care structures. Furthermore, new surgical techniques and non-surgical therapies may contribute to these changes [4, 12–20]. The Organisation of Economic Co-operation and Development (OECD) reports hospital admissions for major lower extremity amputations in adults with diabetes as an indicator for the international comparison of health care quality. For the year 2015 the international variation in amputation rates in adults with diabetes is over 14-fold. Germany ranks after age-sex standardization with 9.2 major amputations per 100,000 population in the highest quarter. The OECD average was 6.4 amputations per 100,000 population [19, 20]. Also a study from the collaboration of vascular registries from Europe and Australasia (VASCUNET) showed elevated amputation rates for Germany [2].

Due to differing data definitions and coding practices between countries the comparability of those results is limited. However, regardless of potential sources of bias inherent in international comparisons, the comparatively high rate of amputations in Germany needs in depth analysis [18, 20].

This study aims to analyze trends in lower limb amputations in Germany over time, as well as outcomes of care. Since amputations of the lower leg are accompanied by a high risk of death [2], in-hospital mortality serves as the main indicator of the quality of care. As several studies reported shifts in the numbers of higher to lower amputation levels the present study defined eight amputation levels in order to display trends in a fine gradation. Based on this detailed differentiation reamputation rates by level during the same hospital stay were analyzed which might indicate the quality of surgical and perioperative health care processes.

## Methods

### Data

The Diagnosis-Related Group Statistics (DRG Statistics) are the German compulsory administrative inpatient discharge data and are provided by the Research Data Centres of the Federal Statistical Office for research purposes. The data cover all German inpatient cases of all types of health insurance in all acute care hospitals. The data consist of information on every inpatient stay, including principal and secondary diagnoses coded by the German adaptation of the International Classification of Diseases (ICD-10-GM), procedures coded according to the German procedure coding system OPS as well as information on sex, age, disposition of patient (discharge status e.g. death or routine) and length of stay. This study analyzed data of the years 2005–2015. Data were accessed via controlled remote data analysis [21].

### Study population

The analysis included all inpatient cases with a lower limb amputation from 2005 to 2015. The cases were identified using the referring procedure codes and categorized into eight groups according to amputation level: hemipelvectomy, hip joint/femoral, knee/lower leg, leg miscellaneous/not further stated, foot complete, mid−/forefoot, toe/foot ray, and foot miscellaneous/not further stated/interior (Additional file 1: Table S1). As the data do not allow for linkage of several hospital stays on the patient level, the unit of analysis are inpatient cases. Patients with several hospital stays were counted once for each hospital stay.

Only the highest level of amputation was used in order to prevent multiple counting of cases with more than one amputation procedure code. Likewise, in cases of bilateral amputations, only the highest amputation was counted.

Cases with reamputations during the same hospital stay were separately analyzed by hierarchizing the first and last amputation level. Reamputations within bilateral amputees were counted only using the highest amputation.

ICD-10-GM codes were used to classify the cases in seven underlying diseases: trauma incl. frostbite/burn etc., tumor, diabetes mellitus without PAD, diabetes mellitus with PAD, PAD without diabetes mellitus, complications/infections/ulcer/gangrene/varicosis/postthrombotic syndrome without diabetes mellitus or PAD, and other diseases (Additional file 1: Table S1).

### Statistics

The number of cases with lower limb amputations was calculated for each year, and descriptive characteristics of the study population were displayed.

For each of the eight amputation levels, the number of cases was related to the general German population [22]. To adjust for demographic effects, the annual number of cases was directly standardized by 5-year age groups and sex according to the demographic structure of the German population in 2005. The standardized number of cases shows how many amputations would have been performed in 2015 under the hypothetical condition of a steady demographic structure since 2005.

In-hospital mortality stratified by amputation level was analyzed by indirect standardization by 5-year age groups and sex according to the demographic structure of inpatients with lower limb amputation in 2005. The standardized mortality ratio (SMR) shows the development of in-hospital mortality over time, independently of changes in the demographic structure of the study population.

Cases with reamputations during the same hospital stay were cumulated over the study period. The first and the last amputation level were compared in a cross tabulation, displaying the frequency of first and last amputation level combinations.

To account for changes in the average length of stay during the observation period, overall in-hospital mortality and overall reamputation rates were additionally computed as events per 1000 in-hospital days (approximated by using the length of stay).

Statistical significance was assessed by 95% confidence intervals. All statistical analyses were conducted using SAS 9.4 (SAS Institute, Cary, NC, USA).

## Results

### Characteristics of inpatient cases with lower limb amputations from 2005 to 2015

The overall number of cases with lower limb amputations rose from 52,096 in 2005 up to 55,595 in 2015 (+ 6.7%).

The number of hospitals providing amputation surgery declined from 1344 to 1109, whereas the median annual number of cases per hospital increased from 25 to 33. The mean length of stay was reduced from 31 to 24 days, and in-hospital mortality decreased from 11.2 to 7.7% (Table 1). Accounting for the reduction in the mean length of stay during the observation period, the number of deaths per 1000 in-hospital days declined from 3.7 to 3.2 deaths (not displayed).

**Table 1:** Characteristics of inpatient cases with lower limb amputations 2005–2015 in Germany

	2005	2007	2009	2011	2013	2015
Number of cases with lower limb amputation N	52,096	51,922	53,646	53,956	55,115	55,595
Number of hospitals N	1,344	1,281	1,249	1,194	1,142	1,109
Median annual case number per hospital (IQR)	25 (11–56)	25 (11–58)	27 (11–63)	28 (11–68)	30 (11–73)	33 (10–75)
Average length of stay	30.6	29.6	27.5	25.9	25.1	24.0
In-hospital mortality N (%)	5,837 (11.2%)	5,462 (10.5%)	5,011 (9.3%)	4,518 (8.4%)	4,482 (8.1%)	4,276 (7.7%)
Women N (%)	19,718 (37.8%)	18,974 (36.5%)	18,571 (34.6%)	17,779 (33.0%)	17,723 (32.2%)	16,971 (30.5%)
Median age (IQR)	72 (64–80)	72 (65–80)	73 (65–80)	73 (65–81)	74 (64–81)	74 (64–81)
Underlying disease N (%)						
Diabetes mellitus without peripheral arterial disease^a^	6,166 (11.8%)	5,557 (10.7%)	5,161 (9.6%)	5,454 (10.1%)	5,386 (9.8%)	6,075 (10.9%)
Diabetes mellitus with peripheral arterial disease^a^	23,887 (45.9%)	24,595 (47.4%)	26,168 (48.8%)	26,286 (48.7%)	26,398 (47.9%)	26,492 (47.6%)
Peripheral arterial disease without diabetes mellitus^a^	12,198 (23.4%)	12,431 (23.9%)	12,519 (23.3%)	12,213 (22.7%)	12,560 (22.8%)	12,272 (22.1%)
Other diseases	9,845 (18.9%)	9,339 (18.0%)	9,798 (18.3%)	10,003 (18.5%)	10,771 (19.5%)	10,756 (19.4%)
Diabetic foot syndrome^b^	–	–	19,674 (36.7%)	22,240 (41.2%)	22,874 (41.5%)	24,371 (43.8%)
Amputation level N (%)						
Hip joint/femoral	13,958 (26.8%)	13,074 (25.2%)	12,342 (23.0%)	11,238 (20.8%)	10,575 (19.2%)	9644 (17.3%)
Knee/lower leg	8,713 (16.7%)	8,149 (15.7%)	7,714 (14.4%)	6,813 (12.6%)	6,515 (11.8%)	6,411 (11.5%)
Foot complete	381 (0.7%)	306 (0.6%)	274 (0.5%)	314 (0.6%)	331 (0.6%)	310 (0.6%)
Mid−/forefoot	6,825 (13.1%)	7,613 (14.7%)	8,505 (15.9%)	9,281 (17.2%)	9,764 (17.7%)	8,378 (15.1%)
Toe/foot ray	21,419 (41.1%)	21,795 (42.0%)	23,941 (44.6%)	25,510 (47.3%)	27,167 (49.3%)	29,153 (52.4%)
Hemipelvectomy	60 (0.1%)	63 (0.1%)	43 (0.1%)	68 (0.1%)	60 (0.1%)	45 (0.1%)
Leg miscellaneous/not further stated	66 (0.1%)	65 (0.1%)	47 (0.1%)	34 (0.1%)	50 (0.1%)	31 (0.1%)
Foot miscellaneous/not further stated or interior^c^	674 (1.3%)	857 (1.7%)	780 (1.5%)	698 (1.3%)	653 (1.2%)	1623 (2.9%)
Revascularization during the same hospital stay N (%)						
Surgery of peripheral arteries	9,094 (17.5%)	9,352 (18.0%)	9,470 (17.7%)	9,125 (16.9%)	9,201 (16.7%)	8,727 (15.7%)
Percutaneous transluminal angioplasty	5,463 (10.5%)	6,730 (13.0%)	8,616 (16.1%)	10,166 (18.8%)	11,622 (21.1%)	12,343 (22.2%)
Reamputation during the same hospital stay N (%)						
Cases with reamputation	6,861 (13.2%)	6,516 (12.5%)	6,090 (11.4%)	5,889 (10.9%)	5,715 (10.4%)	5,646 (10.2%)
In-hospital mortality among cases with reamputation N (%)	997 (14.5%)	921 (14.1%)	819 (13.4%)	717 (12.2%)	718 (12.6%)	663 (11.7%)

IQR: Interquartile Range (25.-75. percentile). For better representation, only every second year is displayed

^a^Principal or secondary diagnosis

^b^Principal or secondary diagnosis. A specific diagnosis code for diabetic foot syndrome was introduced to the ICD-10-GM in 2009

^c^A specific procedure code for interior amputations in the area of the midfoot and footroot bone was introduced to the OPS in 2014 (until 2014 such amputations were presumably coded as foot miscellaneous/not further stated)

The proportion of females decreased from 38% (2005) to 31% in 2015. During the observation period, the median age rose from 72 to 74 years (Table 1).

The percentage of cases with DM as a principal or secondary diagnosis without a coded PAD declined slightly from 12 to 11%. The mixed group of cases with coded DM and PAD represented up to 46% (2005) and 48% (2015). Exclusively coded PAD without DM was coded a little less often in 2015 with 22% than 23% in 2005. Since the introduction of a separate ICD-10 code for diabetic foot syndrome in 2009, this proportion rose to 44% of all cases and 75% (2015) of all cases with DM (Table 1). Over time, the proportion of DM and/or PAD-related underlying diseases was constant at 81–83% of all cases (Additional file 2: Figure S1).

**Fig. 1:**
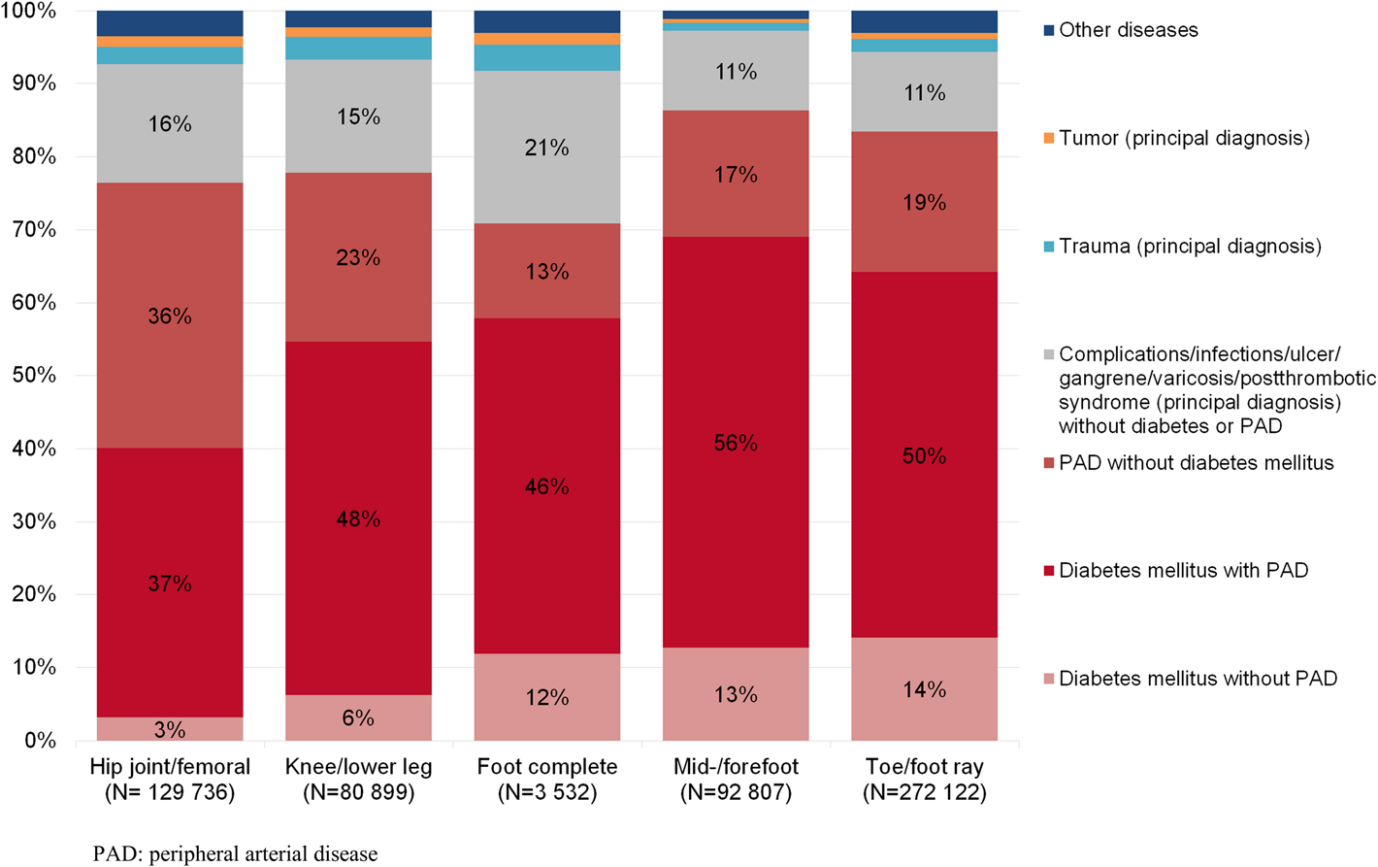
Underlying diseases (%) by amputation level (cases cumulated from 2005 to 2015)

Figure 1 shows the cumulative number of underlying diseases by amputation levels for the observation period. The proportion of DM and/or PAD for the levels of toes/foot ray (83%) and mid−/forefoot (86%) was higher than that for amputation levels above the foot. Within the amputation levels of knee/lower leg, the proportion of DM and/or PAD was 77%, and the proportion of DM and/or PAD diseases for hip joint/femoral amputations was 76% (Fig. 1).

The number of cases with an amputation within the hip joint/femoral decreased between 2005 and 2015 from 13,958 cases (26.8% of all amputations) to 9664 cases (17.3%). Likewise, the number of cases at the levels of knee/lower leg decreased, with 8713 cases (16.7%) in 2005 versus 6411 cases (11.5%) in 2015. In 381 cases (0.7%) and 310 cases (0.6%), the complete foot was amputated. In the area of the mid−/forefoot, a slight increase from 6825 cases (13.1%) to 8378 cases (15.1%) was identified. The most frequent and distinctly rising amputation level was toe/foot ray with 21,419 cases (41.1%) in 2005 and 29,153 cases (52.4%) in 2015 (Table 1).

Cases with surgery of peripheral arteries during the same hospital stay decreased slightly from 17.5% in 2005 to 15.7% of all cases in 2015, whereas the proportion of cases with percutaneous transluminal angioplasty (PTA) during the same stay doubled from 10.5 to 22.2% (Table 1).

The proportion of cases with a reamputation during the same hospital stay declined from 13.2% (6861 cases) in 2005 to 10.2% (5646 cases) in 2015. Accounting for the reduction in the mean length of stay during the observation period, the number of reamputations per 1000 in-hospital days declined from 4.3 to 4.2. The in-hospital mortality of reamputated cases was reduced from 14.5 to 11.7% (Table 1).

### Observed and standardized trends of case numbers

**Fig. 2:**
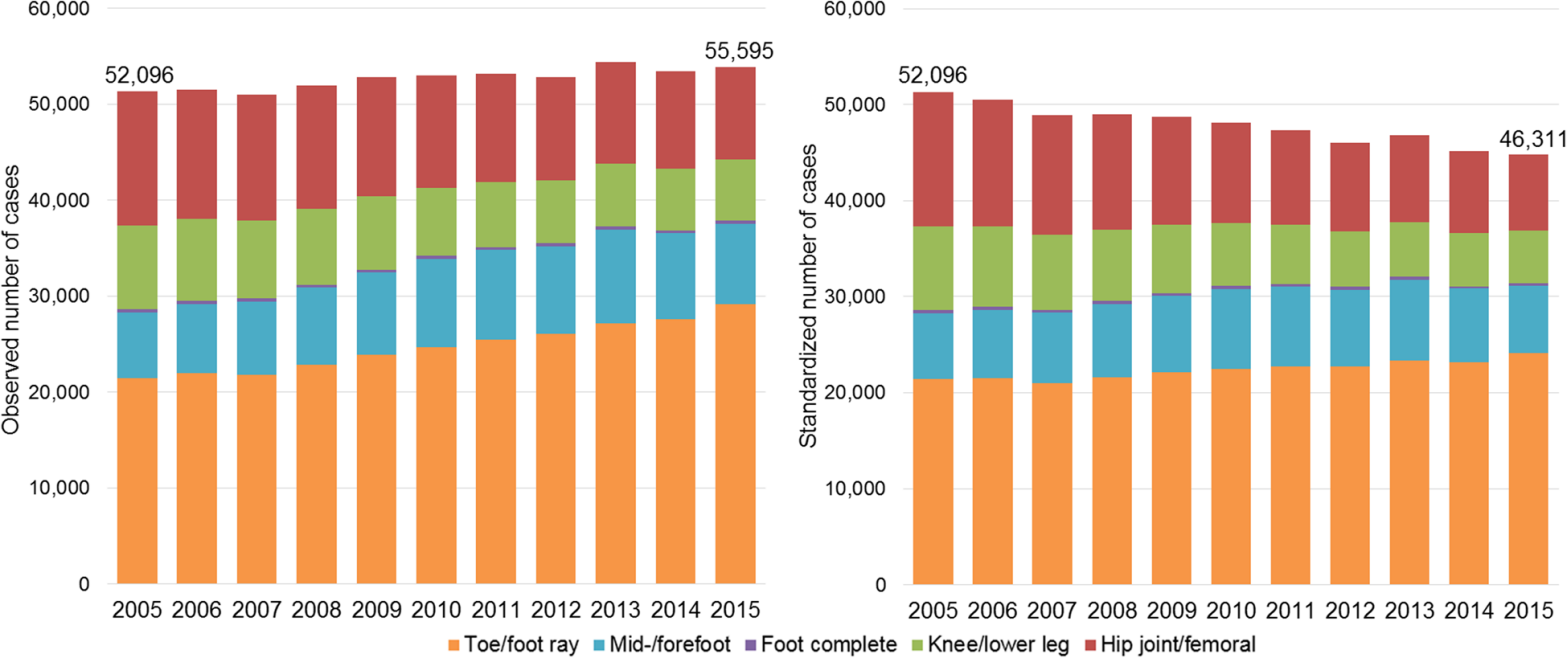
Observed and standardized number of cases and rates per 100,000 persons of lower limb amputations from 2005 to 2015. Left side: Observed number of cases. Right side: Standardized number of cases (reference population of 2005)

Between 2005 and 2015, the observed number of cases increased relatively by 6.7%. The rate per 100,000 persons rose from 63.2 to 66.4 cases. After age-sex standardization to the demographic structure in 2005, a decrease of − 11.1% was revealed (men − 2.6%, women − 25.0%; Fig. 2, Additional file 3: Table S2).

The observed number of cases decreased during the observation period for the levels of hip joint/femoral, knee/lower leg and complete foot. These declines were enhanced with standardization. Within the levels of mid−/forefoot and toe/foot ray, increases were observed. After standardization these increases were slightly reduced (Fig. 2).

### In-hospital mortality by amputation levels

**Fig. 3:**
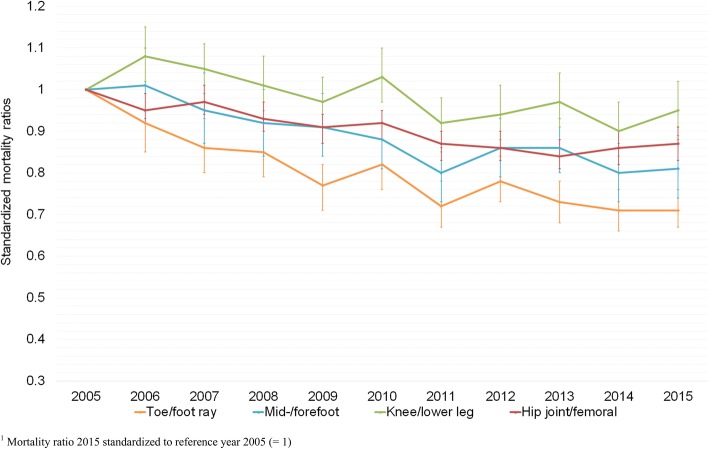
Time trends of standardized mortality ratios^1^ (95% CI) for lower limb amputations

In 2015, 17.4% of the cases with an amputation of the leg and 3.7% of the cases with an amputation of the foot died during the hospital stay. The observed mortality rate and SMR showed reductions in in-hospital mortality over time for the most frequent amputation levels. Compared to the year 2005, in-hospital mortality was significantly reduced for the amputation levels of hip joint/femoral (SMR 0.87); mid−/forefoot (0.81) and toe/foot ray (0.71) (Fig. 3, Additional file 4: Table S3). Likewise, reamputated cases showed a significant decrease in in-hospital mortality with an SMR of 0.80 (Additional file 4: Table S3).

### Reamputations

In the observation period from 2005 until 2015, 67,387 cases with a reamputation during the same hospital stay were identified (Table 2). These cases comprised 151,431 amputation procedures (average of 2.25 procedures per case, not displayed).

**Table 2:** First and last amputation levels of reamputated cases (cases cumulated 2005–2015)

		All cases	Last amputation level								In-hospital mortality		
			Mid−/forefoot	Foot complete	Foot miscellaneous/not further stated/interior	Knee/lower leg	Hip joint/femoral	Leg miscellaneous/not further stated	Hemipel-vectomy	Total reampu-tations	Cases with reamputation	Cases without reamputation	All cases
First amputation level	Toe/foot ray	313,550 (100%)	19,707 (6.29%)	557 (0.18%)	2,556 (0.82%)	10,713 (3.42%)	6,916 (2.21%)	67 (0.02%)	XXX	40,516 (12.92%)	11.67%	3.20%	4.50%
	Mid−/forefoot	85,904 (100%)	–	602 (0.70%)	995 (1.16%)	8,339 (9.71%)	4,688 (5.46%)	34 (0.04%)	XXX	14,658 (17.06%)	14.44%	6.43%	8.43%
	Foot complete	3,359 (100%)	–	–	42 (1.25%)	725 (21.58%)	275 (8.19%)	7 (0.21%)	XXX	1,049 (31.23%)	11.44%	7.71%	9.70%
	Foot miscellaneous/not further stated/interior	10,451 (100%)	–	–	–	528 (5.05%)	309 (2.96%)	XXX	XXX	837 (8.01%)	15.55%	3.57%	4.73%
	Knee/lower leg	69,915 (100%)	–	–	–	–	10,037 (14.36%)	158 (0.23%)	3 (0.00%)	10,198 (14.59%)	19.13%	12.96%	16.07%
	Hip joint/femoral	105,650 (100%)	–	–	–	–	–	80 (0.08%)	49 (0.05%)	129 (0.12%)	31.50%	22.33%	28.78%
	Leg miscellaneous/not further stated	411 (100%)	–	–	–	XXX	XXX	–	XXX	XXX	–	7.54%	8.16%
	Amputations total^a^	589,240 (100%)	19,707 (3.34%)	1,159 (0.20%)	3,593 (0.61%)	20,305 (3.45%)	22,225 (3.77%)	346 (0.06%)	52 (0.01%)	67,387 (11.44%)	13.46%	8.65%	9.20%

xxx: Cell counts < 3 cannot be displayed in terms of data protection law

^a^Without hemipelvectomies (*n* = 546)

Table 2 shows a cross tabulation of the counts and proportions of the last amputation levels in relation to the first amputation level. For example, 12.9% of all amputations within the amputation level of toe/foot ray underwent at least one additional amputation procedure during the same hospital stay. For these cases, the most frequent last amputation level was mid−/forefoot at 6.3%. The in-hospital mortality for reamputated cases (13.5% on average) was distinctly higher than that for cases without reamputation. E.g., for the amputation level of toe/foot ray the in-hospital mortality was 3.2% in cases without reamputation whereas it was 11.7% in cases with a subsequent reamputation at a higher level (Table 2).

## Discussion

The overall observed increase in lower limb amputation cases is attributable to the aging of the German population. After age-sex standardization to the demographic structure of 2005, an overall decrease of − 11% was revealed, which was distinctly stronger in women than in men. The stratified analysis by amputation levels showed that the decreases were induced by higher amputation levels (up to − 43% for hip joint/femoral), whereas the amputation levels of toe/foot ray after standardization still showed a relative increase of + 13%. These trends correspond to the findings of previous studies using German hospital discharge data. Two recent studies reported a decrease for higher and an increase for lower amputation levels. In both studies overall decreases in amputation rates were higher within the female population [14, 16]. However different approaches regarding the inclusion of cases and the definition of amputation levels hamper a direct comparison of results. Nevertheless several international studies also captured decreases in major amputations and increases in minor amputations [2, 6, 12–17, 23] as well as higher decreases in amputation rates within women than men [2, 6, 14, 16, 23].

In the present study the overall amputation rate was much higher in men than in women. A recent German study based on statutory health insurance data found sex differences to hold true for the diabetic as well as the non-diabetic population [23]. This goes in line with results showing no contribution of health care-related factors [24], but biological factors contributing to sex differences in amputation rates [25–27]. However the causes of the sex differences still need further research.

There are several aspects of health care which are discussed to be possible drivers behind the shift in amputation levels [2, 6]. Enhanced surgical amputation techniques and perioperative procedures may hypothetically shift higher amputations to more peripheral levels. However, since the absolute increase in more peripheral amputation levels is much higher than the decrease in higher levels, such a shift can only be accountable for a small proportion of cases. Furthermore improvements in revascularization rates might be responsible to some degree for the decrease in higher amputations. A German study reported that revascularization increased by 33% within hospitalized diabetes patients between 2005 and 2014 in Germany. 78% of that increase was induced by endovascular therapy [15]. Another aspect might be improved ambulatory care for chronic wounds, as guidelines and specialized centers for chronic wound care especially for patients with PAD and DM might have reduced the necessity of amputating in higher levels [15, 28].

Furthermore lifestyle factors as smoking might have influence on amputation trends. In Germany the share of smokers aged 15 years and older decreased from 28.8% in 1992 to 24.5% in 2013 [29]. As higher level amputations are more frequently associated with macroangiopathy and accordingly in this study more frequently associated with PAD, a shrinking smoking population might have contributed to the decrease in higher level amputations. The epidemiological development of diabetes (more frequently associated with microangiopathy) with an increasing prevalence from below 1% in the 1960s to 9.9% in 2015 [9] might explain especially the increase in the number of cases with an amputation of the toe/foot ray, which were more frequently associated with DM in this study. However, there is a broad overlap of PAD and DM. Apart from microangiopathic alterations, DM is considered to be the main risk factor for the development of a PAD [30]. During the observed period there were no changes in the proportions of DM without PAD, DM with PAD or PAD without DM.

In-hospital mortality declined throughout the observation period. A decline was still visible after the number of deaths were related to in-hospital days, aiming to account for the reduction in length of stay during the observation period. Therefore, the observed mortality reductions might indicate an improvement of surgical and perioperative health care. The mean in-hospital mortality for leg amputations was 17.4% and for foot amputations 3.7% in 2015. These results match the in-hospital mortality for major amputations in Germany of 18.5% in 2014 reported in the international VASCUNET study. The lowest in-hospital mortality for major amputations was reported from Finland with 6.1% and the highest from Hungary with 20.9% [2].

The reamputation rate within the same hospital stay, which was analyzed for the first time using complete national data, declined over time and was 10% in 2015. A comparatively high reamputation rate of 17% was seen in cases with a mid−/forefoot amputation. A meta-analysis of reamputations after transmetatarsal amputations estimated a reamputation rate of 28%, including reamputations in subsequent hospital stays [3]. Sjödin et al. (2018) reported reamputations for 9% in their study with 162 Swedish first-ever transfemoral amputation patients [31]. Van Houtum et al. (1996) captured reamputations during the same hospital stay for 13.2% of all diabetes related hospitalizations whereas only 7.2% of the non-diabetic population underwent reamputations [32].

The reamputation rate was attenuated when it was related to in-hospital days, i.e. length of stay. However, reamputations during the same hospital stay are caused by insufficient perfusion of the stump and wound healing complications [31] for which the act of discharging a patient might not be independent. In addition to the reduction in reamputation rates also the in-hospital mortality decreased for these cases. This further emphasizes the hypothesis of improved surgical amputation techniques and perioperative processes.

The strength of this study is the use of complete German hospital discharge data, covering all inpatient cases in German acute care hospitals. As this data is collected for billing purposes accuracy of coding is being validated systematically by insurers. The case-related analysis and hierarchical stratification by amputation level prevents multiple counting of a single case with several coded procedures. However, the data do not permit several inpatient stays or episodes of ambulatory care to be linked. Therefore, analyses of the patient’s history (e.g., preceding revascularizations) or long-term follow-ups (e.g., reamputation in a subsequent hospital stay) are not possible.

## Conclusions

The age-sex standardized number of lower limb amputations declined in Germany, however distinctly stronger in women than in men. The observed decreases of in-hospital mortality as well as of reamputation rates point to improvements of perioperative health care. Despite these indications for improvements, the distinct increase in case numbers at the level of toe/foot ray calls for additional targeted prevention efforts, especially for patients with diabetes.

## Additional files

Additional file 1: **Table S1.** Definition of cases and stratification variables. (DOCX 22 kb)

Additional file 2: **Figure S1.** Yearly proportion of underlying diseases. (PNG 90 kb)

Additional file 3: **Table S2.** Observed and standardized time trends of case numbers from 2005 to 2015. The basis of analysis for age-sex standardized number of cases and rates per 100,000 persons is German population data provided by the German Federal Statistical Office. (DOCX 17 kb)

Additional file 4: **Table S3.** Standardized Mortality Ratios (SMR) by amputation levels.^1^ (DOCX 17 kb)

## Abbreviations

DM: Diabetes mellitus
DRG: Diagnosis related groups
ICD-10-GM: International Classification of Diseases German Modification
OECD: Organisation of Economic Co-operation and Development
OPS: Operation and Procedure Code
PAD: Peripheral arterial diseases
