# Correction to: Amputation rates of the lower limb by amputation level – observational study using German national hospital discharge data from 2005 to 2015

**DOI:** 10.1186/s12913-019-3973-9

**Published:** 2019-03-14

**Authors:** Melissa Spoden, Ulrike Nimptsch, Thomas Mansky

**Affiliations:** 1 Department of Structural Advancement and Quality Management in Health Care, Technische Universität Berlin, Berlin, Germany; 2 Department of Health Care Management, Technische Universität Berlin, H80, Strasse des 17. Juni 135, 10623 Berlin, Germany

**Correction to: BMC Health Serv Res**

**https://doi.org/10.1186/s12913-018-3759-5**

In the original publication of this article [1], some numbers in the below sentence errors in the Results section of the Abstract:

In-hospital mortality of all cases with lower limb amputation fell from 19.8% in 2005 to 17.4% in 2015 (SMR 0.89 [95% CI 0.86; 0.92]).

Other results and interpretation remain unchanged. The numbers are revised in the paragraph below: In-hospital mortality of all cases with lower limb amputation fell from 11.2% in 2005 to 7.7% in 2015.
