# Supplementary material for: Amputation rates of the lower limb by amputation level – observational study using German national hospital discharge data from 2005 to 2015
Source: BMC Health Serv Res. 2019 Jan 6;19:8. doi: 10.1186/s12913-018-3759-5 (PMC6322244; doi:10.1186/s12913-018-3759-5)
Supplement: Supplementary file 1 — Table S1. Definition of cases and stratification variables. (DOCX 22 kb) [file 12913_2018_3759_MOESM1_ESM.docx]

**Table S1** Definition of cases and stratification variables

| **Inpatient cases with amputation of the lower limb** | | |
| --- | --- | --- |
|  | **German procedure coding system (OPS, Operationen- und Prozedurenschlüssel)** | |
|  | **Inclusion criteria** | **Exclusion criteria** |
| All amputations of the lower limb | 5864, 5865 |  |
| **Amputation levels leg (major)** | |  |
| Hemipelvectomy complete/incomplete | 58640, 58641 |  |
| Hip joint/femoral | 58642, 58643, 58644, 58645 | 58640, 58641 |
| Knee/lower leg | 58646, 58647, 58648, 58649, 5864a | 58640, 58641, 58642, 58643, 58644, 58645 |
| Leg miscellaneous/not further stated | 5864x, 5864y | 58640, 58641, 58642, 58643, 58644, 58645, 58646, 58647, 58648, 58649, 5864a |
| **Amputation levels foot (minor)** | |  |
| Foot complete | 58650, 58651, 58652, 58653 | 58640, 58641, 58642, 58643, 58644, 58645, 58646, 58647, 58648, 58649, 5864a, 5864x, 5864y |
| Mid-/forefoot | 58654, 58655, 58656 | 58640, 58641, 58642, 58643, 58644, 58645, 58646, 58647, 58648, 58649, 5864a, 5864x, 5864y, 58650, 58651, 58652, 58653 |
| Toe/footray | 58657, 58658 | 58640, 58641, 58642, 58643, 58644, 58645, 58646, 58647, 58648, 58649, 5864a, 5864x, 5864y, 58650, 58651, 58652, 58653, 58654, 58655, 58656 |
| Foot miscellaneous/not further stated/interior | 58659, 5865x, 5865y | 58640, 58641, 58642, 58643, 58644, 58645, 58646, 58647, 58648, 58649, 5864a, 5864x, 5864y, 58650, 58651, 58652, 58653, 58654, 58655, 58656, 58657, 58658 |
| **Underlying diseases** | | |
|  | **International Classification of Diseases 10th Revision, German Modification ICD-10-GM** | |
|  | **Inclusion criteria** | **Exclusion criteria** |
| Trauma incl. frostbite/burn etc. | PD:  S0, S1, S2, S3, S4, S5, S6, S7, S8, S9, T0, T10, T11, T12, T13, T14, T2, T30, T31, T32, T33, T34, T35, T66, T67, T68, T69, T70, T71, T72, T73, T74 , T75, T79, T89, T9 |  |
| Tumor | PD:  C, D0 |  |
| Diabetes mellitus without peripheral arterial disease (PAD) | PD or SD:  E10, E11, E12, E13, E14 | PD or SD:  S0, S1, S2, S3, S4, S5, S6, S7, S8, S9, T0, T10, T11, T12, T13, T14, T2, T30, T31, T32, T33, T34, T35, T66, T67, T68, T69, T70, T71, T72, T73, T74 , T75, T79, T89, T9, C, D0, I702, I709 |
| PAD without diabetes mellitus | PD or SD:  I702, I709 | PD or SD:  S0, S1, S2, S3, S4, S5, S6, S7, S8, S9, T0, T10, T11, T12, T13, T14, T2, T30, T31, T32, T33, T34, T35, T66, T67, T68, T69, T70, T71, T72, T73, T74 , T75, T79, T89, T9, C, D0, E10, E11, E12, E13, E14 |
| Diabetes mellitus with PAD | PD or SD:  E10, E11, E12, E13, E14  &  PD or SD:  I702, I709 | PD:  S0, S1, S2, S3, S4, S5, S6, S7, S8, S9, T0, T10, T11, T12, T13, T14, T2, T30, T31, T32, T33, T34, T35, T66, T67, T68, T69, T70, T71, T72, T73, T74 , T75, T79, T89, T9, C, D0 |
| Complications/infections/ulcer/gangrene/varicosis/postthrombotic syndrome without diabetes mellitus or PAD | PD:  T823, T824, T825, T827, T828, T829, T84, T874, T875, T876, M00, M86, M900, M901, M902, M87, M895, M903, M904, M905, A46, A480, L0302, L0311, R02, L97, L984, L8927, L8928, L8937, L8938, I8721, I830, I831, I832, I870, A40, A41, R572, R650, R651 | SD:  E10, E11, E12, E13, E14, I702, I709 |
| Diabetic foot syndrome | PD or SD:  E1074, E1075, E1174, E1175 |  |
| **Ongoing Table S1** Definition of cases and stratification variables | | |
| **Revascularization during the same hospital stay** | | |
|  | **German procedure coding system (OPS, Operationen- und Prozedurenschlüssel)** | |
|  | **Inclusion criteria** | **Exclusion criteria** |
| Surgery of peripheral arteries | 53805, 53807, 53808, 53815, 53817, 53818, 538253, 538254, 538255, 53825x, 53827,  53828, 538352, 538353, 538354, 538355, 53835x, 53837, 53838, 539333, 539335,  539336, 539338, 53933x, 539341, 539342, 539343, 539344, 539345, 539346, 539347, 53934x, 53935, 53936, 53937, 539552, 539553, 539554, 539555, 53955x, 53957, 53958, 53965, 53967, 53968, 53975, 53977, 53978 |  |
| Percutaneous transluminal angioplasty (PTA) | 883609, 88360a, 88360b, 88360c, 883619, 88361a, 88361b, 88361c, 883629, 88362a, 88362b, 88362c, 883639, 88363a, 88363b, 88363c, 883679, 88367a, 88367b, 88367c, 883689, 88368a, 88368b, 88368c, 8836d9, 8836da, 8836db, 8836dc, 8836e9, 8836ea, 8836eb, 8836ec, 8836f9, 8836fa, 8836fb,  8836fc, 8836g9, 8836ga, 8836gb, 8836gc, 8836h9, 8836ha, 8836hb, 8836hc, 8836j9, 8836ja, 8836jb, 8836jc, 8836p9, 8836pa,  8836pb, 8836pc, 8836wb, 8836wc, 8836x9, 8836xa, 8836xb, 8836xc, 884009, 88400a, 88400b, 88400c, 884019, 88401a, 88401b, 88401c, 884029, 88402a, 88402b, 88402c, 884039, 88403a, 88403b, 88403c, 884049, 88404a, 88404b, 88404c, 884059, 88405a, 88405b, 88405c, 884109, 88410a, 88410b, 88410c, 884119, 88411a, 88411b, 88411c, 884129, 88412a, 88412b, 88412c, 884139, 88413a, 88413b, 88413c, 884149, 88414a, 88414b, 88414c, 884159, 88415a, 88415b, 88415c, 884209, 88420a, 88420b, 88420c, 884219, 88421a, 88421b, 88421c, 884229, 88422a, 88422b, 88422c, 884239, 88423a, 88423b, 88423c, 884249, 88424a, 88424b, 88424c, 884259, 88425a, 88425b, 88425c, 884309, 88430a, 88430b, 88430c, 884319, 88431a, 88431b, 88431c, 884329, 88432a, 88432b, 88432c, 884339, 88433a, 88433b, 88433c, 884349, 88434a, 88434b, 88434c, 884359, 88435a, 88435b, 88435c, 88440c, 88441c, 88442c, 88443c, 88444c, 88445c, 884509, 88450a, 88450b, 88450c, 884519, 88451a, 88451b, 88451c, 884609, 88460a, 88460b, 88460c, 884619, 88461a, 88461b, 88461c, 884809, 88480a, 88480b, 88480c, 884819, 88481a, 88481b, 88481c, 884829, 88482a, 88482b, 88482c, 884839, 88483a, 88483b, 88483c, 884849, 88484a, 88484b, 88484c, 884859, 88485a, 88485b, 88485c, 884909, 88490a, 88490b, 88490c, 884919, 88491a, 88491b, 88491c, 884a09, 884a0a, 884a0b, 884a0c, 884a19, 884a1a, 884a1b, 884a1c, 884b09, 884b0a, 884b0b, 884b0c, 884b29, 884b2a, 884b2b, 884b2c, 884b39, 884b3a, 884b3b, 884b3c, 884b49, 884b4a, 884b4b, 884b4c, 884b59, 884b5a, 884b5b, 884b5c, 884b69, 884b6a, 884b6b, 884b6c |  |

PD: principal diagnosis

SD: secondary diagnosis

PAD: peripheral arterial disease
