# Supplementary figures and images for: Amputation rates of the lower limb by amputation level – observational study using German national hospital discharge data from 2005 to 2015
Source: BMC Health Serv Res. 2019 Jan 6;19:8. doi: 10.1186/s12913-018-3759-5 (PMC6322244; doi:10.1186/s12913-018-3759-5)

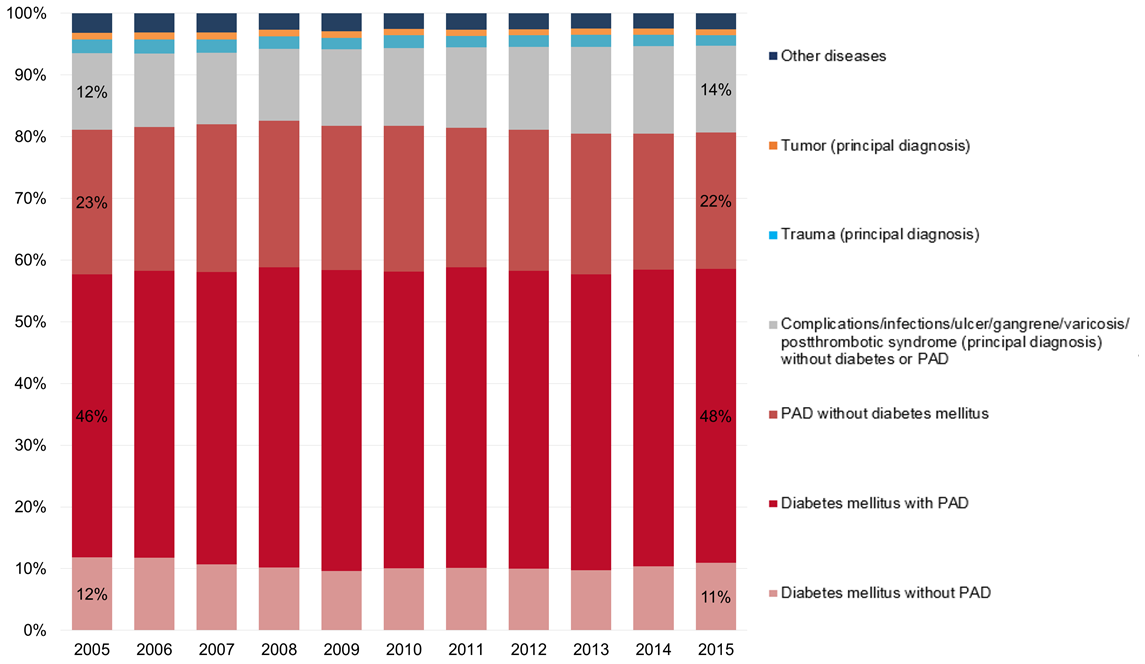

Supplement: Supplementary file 2 — Figure S1. Yearly proportion of underlying diseases. (PNG 90 kb) [file 12913_2018_3759_MOESM2_ESM.png]
