# Supplementary material for: Amputation rates of the lower limb by amputation level – observational study using German national hospital discharge data from 2005 to 2015
Source: BMC Health Serv Res. 2019 Jan 6;19:8. doi: 10.1186/s12913-018-3759-5 (PMC6322244; doi:10.1186/s12913-018-3759-5)
Supplement: Supplementary file 3 — Table S2. Observed and standardized time trends of case numbers from 2005 to 2015. The basis of analysis for age-sex standardized number of cases and rates per 100,000 persons is German population data provided by the German Federal Statistical Office. (DOCX 17 kb) [file 12913_2018_3759_MOESM3_ESM.docx]

**Table S2** Observed and standardized time trends of case numbers from 2005 to 2015

|  | **Number of cases** | | | **Relative change in case numbers from 2005 to 2015** | | **Rate per 100 000 persons** | | |
| --- | --- | --- | --- | --- | --- | --- | --- | --- |
|  | Observed  2005 | Observed 2015 | Standardized 2015 | Observed  relative change | Standardized relative change | Observed 2005 | Observed 2015 | Standardized 2015 |
|  |  |  |  |  |  |  |  |  |
|  |  |  |  |  |  |  |  |  |
|  |  |  |  |  |  |  |  |  |
| Overall | 52 096 | 55 595 | 46 311 | +6.7% | -11.1% | 63.19 | 66.44 | 56.18 |
| **Sex** |  |  |  |  |  |  |  |  |
| Men | 32 378 | 38 624 | 31 525 | +19.3% | -2.6% | 80.26 | 93.09 | 78.15 |
| Women | 19 718 | 16 971 | 14 786 | -13.9% | -25.0% | 46.84 | 40.23 | 35.12 |
|  |  |  |  |  |  |  |  |  |
| **Amputation levels** |  |  |  |  |  |  |  |  |
| Hip joint/femoral | 13 958 | 9 644 | 7 962 | -30.9% | -43.0% | 16.93 | 11.53 | 9.66 |
| Knee/lower leg | 8 713 | 6 411 | 5 494 | -26.4% | -36.9% | 10.57 | 7.66 | 6.66 |
| Foot complete | 381 | 310 | 272 | -18.6% | -28.6% | 0.46 | 0.37 | 0.33 |
| Mid-/forefoot | 6 825 | 8 378 | 6 974 | +22.8% | +2.2% | 8.28 | 10.01 | 8.46 |
| Toe/foot ray | 21 419 | 29 153 | 24 161 | +36.1% | +12.8% | 25.98 | 34.84 | 29.31 |
| Others* | 800 | 1 699 | 1 447 | +112.4% | +80.9% | 0.97 | 2.03 | 1.76 |

The basis of analysis for age-sex standardized number of cases and rates per 100 000 persons is German population data provided by the German Federal Statistical Office.

* Hemipelvectomy complete/incomplete, leg miscellaneous/not further stated, foot miscellaneous/not further stated/interior
