# Supplementary material for: Amputation rates of the lower limb by amputation level – observational study using German national hospital discharge data from 2005 to 2015
Source: BMC Health Serv Res. 2019 Jan 6;19:8. doi: 10.1186/s12913-018-3759-5 (PMC6322244; doi:10.1186/s12913-018-3759-5)
Supplement: Supplementary file 4 — Table S3. Standardized Mortality Ratios (SMR) by amputation levels.1 (DOCX 17 kb) [file 12913_2018_3759_MOESM4_ESM.docx]

**Table S3** Standardized Mortality Ratios (SMR) by amputation levels

|  | **In-hospital mortality** | | **Number of cases** | **Number of cases** | **SMR [95% CI]^1^** |
| --- | --- | --- | --- | --- | --- |
|  | **2005** | **2015** | **2005** | **2015** | **2015 to 2005** |
| **All leg amputations** | **19.8%** | **17.4%** | **22 797** | **16 131** | **0.89 [0.86; 0.92]** |
| Hemipelvectomy | 10.0% | 11.1% | 60 | 45 | 1.15 [0.37; 2.69] |
| Hip joint/femoral | 24.2% | 21.1% | 13 958 | 9 644 | 0.87 [0.83; 0.91] |
| Knee/lower leg | 12.8% | 12.0% | 8 713 | 6 411 | 0.95 [0.89; 1.02] |
| Leg miscellaneous/not further stated | 15.2% | 9.7% | 66 | 31 | 0.66 [0.13; 1.93] |
|  |  |  |  |  |  |
| **All foot amputations** | **4.5%** | **3.7%** | **29 299** | **39 154** | **0.73 [0.69; 0.76]** |
| Foot complete | 7.9% | 9.7% | 381 | 310 | 1.19 [0.80; 1.69] |
| Mid-/forefoot | 7.2% | 6.4% | 6 825 | 8 378 | 0.81 [0.74; 0.88] |
| Toe/foot ray | 3.6% | 2.9% | 21 419 | 29 153 | 0.71 [0.67; 0.76] |
| Foot miscellaneous/not further stated/interior^2^ | 5.6% | 2.6% | 674 | 1 623 | 0.47 [0.34; 0.63] |
|  |  |  |  |  |  |
| **Reamputated cases** | **14.5%** | **11.7%** | **6 861** | **5 646** | **0.80 [0.73; 0.86]** |

^1^ Standardized to the demographic structure of 2005 (2005=1).

^2^ Limited comparability due to changes of OPS codes in 2014.
